# “As du Coeur” study: a randomized controlled trial on quality of life impact and cost effectiveness of a physical activity program in patients with cardiovascular disease

**DOI:** 10.1186/s12872-018-0973-3

**Published:** 2018-12-06

**Authors:** Laurent Bailly, Philippe Mossé, Stéphane Diagana, Marion Fournier, Fabienne d’Arripe-Longueville, Odile Diagana, Jocelyn Gal, Jean Grebet, Mario Moncada, Jean-Jacques Domerego, Rémi Radel, Roxane Fabre, Alain Fuch, Christian Pradier

**Affiliations:** 1 Département de Santé Publique, Centre Hospitalier Universitaire de Nice (Public Health Department University Hospital of Nice), F-06202 Nice, France; 2 Université Côte d’Azur, LAMHESS, Nice, France; 3 LEST, Aix-Marseille Université, CNRS, Aix en Provence, France; 4 Diagana Sport Santé, Nice, France; 5 Azur Sport Santé, Nice, France; 6 Epidemiology and Biostatistics Unit, Centre Antoine Lacassagne, Nice, France; 7 Sécurité Sociale des Indépendants, Nice, France; 8 Hôpital Privé Gériatrique les Sources, Nice, France; 9 EA Cobtek, University of Nice Sophia-Antipolis, Nice, France; 10 Département de Santé Publique, CHU Nice, Hôpital Archet 1. Niveau1 151 Route Saint Antoine de Ginestière CS 23079, 06202 Nice Cedex 3, France

**Keywords:** Cost-effectiveness, Physical activity, Cardiovascular disease, Quality of life, Health care costs

## Abstract

**Background:**

Physical activity programs (PAP) in patients with cardiovascular disease require evidence of cost-utility. To assess improvement in health-related quality of life (QoL) and reduction of health care consumption of patients following PAP, a randomized trial was used.

**Methods:**

Patients from a health insurance company who had experienced coronary artery disease or moderate heart failure were invited to participate (*N* = 1891). Positive responders (*N* = 50) were randomly assigned to a progressively autonomous physical activity (PAPA) program or to a standard supervised physical activity (SPA) program. The SPA group had two supervised sessions per week over 5 months. PAPA group had one session per week and support to aid habit formation (written tips, exercise program, phone call). To measure health-related quality of life EQ-5D utility score were used, before intervention, 6 months (T6) and 1 year later. Health care costs were provided from reimbursement databases.

**Results:**

Mobility, usual activities and discomfort improved significantly in both group (T6). One year later, EQ-5D utility score was improved in the PAPA group only. Total health care consumption in the intervention group decreased, from a mean of 4097 euros per year before intervention to 2877 euros per year after (*p* = 0.05), compared to a health care consumption of 4087 euros and 4180 euros per year, in the total population of patients (*N* = 1891) from the health insurance company. The incremental cost effectiveness ratio was 10,928 euros per QALYs.

**Conclusion:**

A physical activity program is cost-effective in providing a better quality of life and reducing health care consumption in cardiovascular patients.

**Trial registration:**

ISRCTN77313697, retrospectively registered on 20 November 2015.

**Electronic supplementary material:**

The online version of this article (10.1186/s12872-018-0973-3) contains supplementary material, which is available to authorized users.

## Background

Cardiovascular disease is a burden in France, as in many western countries [1]. Physical activity in patients with cardiovascular disease has been shown to improve exercise capacity and health-related quality of life and to reduce hospital admissions [2]. The aim is to promote the adoption and maintenance [3] of healthful behaviors in cardiovascular patients, notably by supervised physical activity (PA) programs. However, many of those patients do not maintain regular physical activity [4, 5] after program completion. Recent studies have identified habit as the main predictor of physical activity maintenance [6, 7]. Habit theory assumes that habits are context-dependent, and this might explain why physical activity is difficult to maintain in a patient’s “real” living environment after cardiac rehabilitation. While a supervised physical activity program has the advantage of providing a safe environment and social exchange, it cannot reproduce an everyday life context that is characterized by sedentary habits. Thus, a transition between a cardiac rehabilitation program and home-based exercise appears necessary. The “As du Coeur” study is a French monocentric, two-arm, randomized study to compare a standard supervised physical activity program (SPA) and a progressively autonomous physical activity (PAPA) intervention in cardiovascular patients [8]. The first objective of the “As du Coeur study” was to compare the two interventions on physical activity maintenance [9]. The second objective was to establish the cost effectiveness of the program.

Some studies have already shown that cardiac rehabilitation programs significantly reduce health care costs [10–12]. Despite this evidence, health care consumption reduction cannot be directly applied to each country as access to the health care systems and health care consumption vary from one place to another. Therefore, it is important to provide economic evaluation for a physical activity program specifically adapted to cardiovascular patients in France. As a result, the objectives of this study were to establish the cost effectiveness of the French physical activity program “As du Coeur”, to evaluate the efficiency of a progressively autonomous versus a standard supervised physical activity program on quality of life improvement, and to ascertain the reduction in health care consumption for cardiovascular patients.

## Methods

This trial, registered as ISRCTN77313697 and approved by the Nice University Hospital ethics committee in 2014 (N° ID – RCB: 2014-A01559–38), is a French monocentric, two-arm, randomized study to compare a standard supervised physical activity program and a progressively autonomous physical activity intervention in cardiovascular patients. The “As du Coeur” study design, settings and randomization process have already been detailed elsewhere [8, 9].

### Consent

Participants were asked to sign a consent form, following a complete explanation of the study, provided in person. They were encouraged to ask questions at this time and were assured of their right not to participate in the study or to withdraw from it at any moment without giving a reason.

### Participants

Cardiovascular patients were identified from the reimbursement database of a health insurance company. Participants had to be over 18 years old, registered with coronary syndrome or heart failure and considered as sedentary according to a brief physical activity assessment [13]. Each participant had to be able to walk at least 250 m in the six-minute walk test.

The intervention program started in January 2015. Patients of the progressively autonomous physical activity group had two supervised sessions per week for the first 2.5 months, and only one supervised session per week for the last 2.5 months. The supervised physical activity group had two supervised sessions per week over 5 months. The intensity levels were defined in accordance with ACSM guidelines [14]. Autonomous practice was promoted in the progressively autonomous physical activity group with support to aid habit formation: written tips, an exercise program and a calendar [6]. They were called every 2 weeks for a conversation with a researcher trained in habit formation theory.

### Quality of life and cost effectiveness evaluation

Health status was evaluated with the EQ-5D tool [15], a simple, generic measure of health-related quality of life for clinical and economic appraisal [16]. The EQ-5D-3 L descriptive system includes five dimensions and each has three levels: no problems, some problems and extreme problems (Additional file 1). Responses to the questionnaire were mapped to utility weight based on values derived from a representative sample of the French population [17, 18]. In our population, the value of this utility score ranges from 1, corresponding to perfect health status, to 0.194.

To estimate life expectancy gain, we used the six-minute walk test, recognized as a valuable independent predictive factor for prognosis in numerous diseases [19–22]. Based on recent published data [19, 20, 23] and selecting the highest threshold of 468 m, our assumption was that a walking distance measured above 468 m in the six-minute walk test could be considered as a 3 years life expectancy gain in cardiovascular patients.

Program costs were assessed by the time spent by the various stakeholders, cost for patients, and the cost of materials. The average program cost per patient was considered as incremental cost. Cost utility was then calculated as incremental cost per Quality Adjusted Life Years (QALYs) gained per patient [24] at 1 year corresponding to average cost per patient of the physical activity program weighted by EQ-5D utility score gain at 1 year.

Health care costs were compared with the total population of cardiovascular patients identified from the reimbursement database. Health care costs for intervention and total population were estimated using costs published by the insurance company.

### Statistical analysis

Continuous variables were expressed as mean and standard deviation (SD) and were compared by Student’s *t* test. Categorical parameters were compared by the Pearson chi-square test or the Fisher exact test. For health care cost comparison and quality of life scores, a normal distribution assumption was verified by graphical tools and statistical tests, i.e., the Shapiro-Wilk and Kolmogorov-Smirnov tests. In the case of a normal distribution assumption, Student’s *t* test or a paired *t* test was used as appropriate, otherwise the Wilcoxon test was used. For the case of exceptional health care consumption and to avoid biased means, costs above a threshold, fixed to 15 times the median value in the total population, were not taken into consideration.

## Results

Fifty patients were randomly assigned to either the progressively autonomous physical activity (PAPA) program or the supervised physical activity (SPA) program. Five patients were lost to follow-up. Baseline characteristics of the 45 participants are presented in Table 1. There was no significant difference of age, sex ratio, body mass index, or EQ-5D utility score between PAPA patients and SPA patients. Some differences appeared for the six-minute walk test, as PAPA patients walked a mean distance of 600 m against 524 m for SPA patients. Compared to the studies using the six-minute walk test as a prognostic factor [20, 22, 23], at baseline our participants walked a distance corresponding to the highest quartile, similar to those observed in a French population of patients [25] at the end of a cardiac rehabilitation program after an acute coronary syndrome, i.e. 510 to 620 m. At the same time, there were more patients with moderate heart failure and with anticoagulant treatment, in the supervised group. In our population study, most of our patients had a coronary artery disease history without heart failure and were classified in class I or II of the New York Heart Association (NYHA).

**Table 1:** Baseline characteristics

	Progressively autonomous group (*n* = 22)	Supervised group (*n* = 23)	p
Age, mean ± SD	62.5 ± 10.7	63.5 ± 8.1	0.64
Sex, n (%)			0.58
Male	21 (95.5)	21 (91.3)	
Female	1 (4.5)	2 (8.7)	
Weight, kg ± SD	82.4 ± 13.7	86.8 ± 12.3	0.27
BMI, kg/m^2^ ± SD	27.1 ± 4.0	29.5 ± 4.0	0.07
AC, cm ± SD	103.5 ± 12.3	106.0 ± 8.7	0.45
EQ5D, mean ± SD	0.828 ± 0.181	0.791 ± 0.161	0.48
6MWT distance, meters ±SD	600.8 ± 84.2	524.0 ± 88.1	0.0064
NYHA, n (%)			
I	11 (52.4)	6 (27.3)	0.32
II	7 (33.3)	11 (50.0)	
III	3 (14.3)	4 (18.2)	
IV	–	1 (4.5)	
Past medical history, n (%)			
Diabetes	4 (18.2)	3 (14.3)	0.73
Hypertension	6 (27.3)	6 (26.1)	0.79
Hyperlipidemia	16 (72.7)	15 (65.2)	0.59
Moderate heart failure	1 (4.5)	7 (33.3)	0.0212
Myocardial infarction	5 (22.7)	9 (40.9)	0.19
Revascularization, n (%)			
Angioplasty	15 (68.2)	13 (56.5)	0.42
CABG	3 (13.6)	4 (19.0)	0.70
Cardiac function, n (%)			
LVEF < 50%	1 (4.8)	3 (14.3)	0.13
LVEF > 50%	20 (95.2)	18 (85.7)	
Current smokers, n (%)	4 (18.2)	3 (14.3)	0.73
Drugs, n (%)			
Antiplatelet drugs	19 (86.4)	16 (69.6)	0.17
Anticoagulants	**–**	4 (19.0)	0.0485
Beta-blockers	11 (50.0)	11 (47.8)	0.88

*Abbreviations*: *AC* Abdominal circumference, *BMI* Body Mass Index, *CABG* Coronary artery bypass graft, *LVEF* Left ventricular ejection fraction, *6MWT* Six-minute walk test, *SD* Standard deviation

### Quality of life and cost-utility

At the end of the intervention, an improvement was observed for health-related quality of life problems measured with the EQ-5D questionnaire. Participants with some or extreme problems decreased significantly compared to baseline, for mobility (from 20.5 to 5.1%), usual activities (from 15.9 to 0%), and pain or discomfort (from 79.5 to 71.8%). The EQ-5D utility score mean was 0.828 in the PAPA group at baseline and 0.891 after intervention and 0.882 at 1 year, corresponding to a gain of 0.054. In the supervised group, the EQ-5D utility score mean was 0.791 at baseline and increased to 0.805 after the intervention, but it decreased to 0.779 at 1 year.

There was an improvement in the walking distance in the six-minute walk test from 561 m at baseline against 605 m at T6 (*p* < 0.001). Considering the threshold distance of 468 m in the six-minute walk test, 38 participants were above it and seven under. The EQ-5D utility score mean was 0.808 in the above 468 m group before intervention and 0.863 after the physical activity program, corresponding to a gain of 0.055. At 1 year, there was still a gain in the group walking more than 468 m as the EQ-5D utility score mean was 0.847, i.e., + 0.039. On the contrary, in the group walking less than 468 m, the EQ-5D utility score mean was 0.812 before and 0.763 at the end of the intervention, and 0.724 at 1 year.

The cost of the physical activity program per patient was 1279 euros for 5 months. Considering the threshold of a walking distance of 468 m in the six-minute walk test, an EQ-5D utility score gain of plus 0.039 and a life expectancy of 3 years for the 38 participants concerned, the incremental cost-effectiveness ratio was 10,928 € per QALYs. The average incremental cost per patient for the PAPA group was 145 euros, corresponding essentially to 3 one-hour phone sessions with a coach. This incremental cost was divided by the baseline-adjusted incremental QALYs. In the PAPA group the incremental cost-effectiveness ratio was 2685 euros per QALYs at 1 year.

### Health care costs

The means of health care costs per year in the total population and in the intervention group, corresponding to the PAPA group and the SPA group, are presented in Table 2. In the total population, the total cost per person was 4087 euros before intervention and 4180 euros during the year of intervention. In the intervention group, while the total health care cost was similar before intervention (i.e., 4097 euros), it significantly (*p* = 0.05) decreased to 2877 euros during the year of intervention. This corresponded to a reduction of nearly 30%. Considering each item separately, we notice a clear decrease of cardiac medications and cardiac visits in the total population and in the intervention group. On the contrary, biological tests and nurse consultations decreased in the intervention group and increased in the total population.

**Table 2:** Health care costs means in euros per year and per person

	Total population					Intervention group		
	2013–2014 (*N* = 1891)	2015 (*N* = 1891)	p	%^a^	2013–2014 (*N* = 43)	2015 (*N* = 43)	p	%^a^
Hospital	2447.5	2169.0	0.61	−11,4%	1970.1	872.2	0.12	−55,7%
Cardiac medications	431.4	339.7	< 0.001	−21,3%	523.3	398.3	< 0.001	−23,9%
Other drugs	751.7	678.9	< 0.001	−9,7%	694.1	643.7	0.25	−7,3%
Cardiac visits	99.4	85.9	< 0.001	−13,6%	91.7	71.7	0.0168	−21,8%
General practitioner	244.1	277.3	< 0.001	+ 13,6%	210.7	239.5	0.08	+ 13,7%
Specialist visits	135.2	141.6	0.35	+ 4,7%	125.5	137.4	0.79	+ 9,5%
Biological tests	175.8	224.3	< 0.001	+ 27,6%	207.7	162.4	0.13	−21,8%
Nurse consultations	205.6	219.3	0.51	+ 6,7%	268.5	201.6	0.0216	−24,9%
Investigations	526.0	537.1	0.92	+ 2,1%	696.4	668.1	0.84	−4,1%
Total	4087.3 €	4179.9 €	0.41	+ 2,3%	4096.7 €	2877.0 €	0.05	−29,8%

^a^ Health care consumption evolution between years 2013–2014 and year 2015

## Discussion

Our study suggests a significant reduction of health care consumption after a physical activity program in patients with cardiovascular disease. It also confirmed our hypothesis that an intervention for people with cardiovascular disease that promotes habit formation in an everyday life context, will result in better maintenance of quality of life.

The comparison of the progressively autonomous physical activity (PAPA) against the standard supervised physical activity (SPA) programs, using the EQ-5D utility score shows that health-related quality of life maintenance at 1 year was observed only in the PAPA group. Our physical activity program appeared to be cost effective, in providing a better quality of life in cardiovascular patients able to walk more than 468 m in the six-minute walk test. The EQ-5D utility score means were improved in participants walking more than 468 m in the six-minute walk test, from 0.808 at baseline to 0.863 after the physical activity program, to 0.847 at 1 year. The gains of plus 0.055 after the physical activity program and plus 0.039 at 1 year are in line with those minimum clinical differences. Goldsmith et al. [26] studied the relationship between measures used in studies of cardiac disease and the EQ-5D index. The authors concluded that the minimum clinically important difference of a one-minute increase in treadmill exercise time was associated with a 0.019 increase in the EQ-5D index, and a 10 unit increase in Seattle Angina Questionnaire (SAQ) scales was associated with an increase between 0.04 and 0.07 in EQ-5D. Recent large trials in patients with heart failure have shown that health-related quality of life values were independently associated with long-term cardiovascular morbidity and mortality, and notably EQ-5D utility score [27, 28]. Ambrosy et al. have shown a 20 % increased risk of death or hospitalization for a decrease of 0.1 units in baseline EQ-5D utility score.

The EQ-5D utility score means decreased in the seven participants walking less than 468 m in the six-minute walk test. For those patients, the physical activity program was clearly not cost effective, suggesting the need to raise the usually retained threshold, 250 m, from a cost effectiveness point of view. Gusi et al. [29] have already shown that the baseline level of the six-minute walk test could predict the magnitude of quality of life changes.

The incremental cost effectiveness ratio was of 10,928 euros per Quality Adjusted Life Years, which is under the threshold usually retained [30, 31] for a gain of one QALY. Miller et al. [32] reported median values per QALY by category of cardiovascular disease studies, published between 2000 and 2011. Values range from 22,113 to 49,637 dollars (19,485 to 43,743 euros), respectively for coronary heart disease and congestive heart failure treatment.

At the same time, total health care consumption showed a clear decrease after the intervention, from 4097 euros to 2877 euros, whereas there was no difference in consumption among the total population of patients invited to participate in this physical activity program, i.e., from 4087 euros to 4180 euros. The total health care consumption mean difference, i.e. 1220 euros, corresponds to the cost of the physical activity program for one patient, i.e. 1279 euros.

We have shown that patients following a progressively autonomous physical activity program had a better EQ-5D utility score mean at 1 year from the intervention, compared to patients with standard supervised physical activity program. Respectively, the index values were 0.882 in the progressively autonomous group and 0.779 in the supervised group at 1 year. The supplementary cost in the progressively autonomous group was essentially due to phone contact with the group coach for follow-up. The phone call for quality of life improvement at 1 year is well worth the cost, as the call cost only 145 euros per patient out of an average total cost of 1279 euros.

Our study has some limitations. Although the hypothesis of our study for health care consumption reduction has been confirmed, the relative low number of participants and the voluntary-based recruitment, for a five-month long physical activity program without interruption, leads us to consider prudently the extrapolation to other populations of the total health care consumption reduction observed in our study.

Another limit of our study could be that the estimation of life expectancy gain, after a physical activity program in cardiovascular patients, is grounded on hypothesis rather than on direct observation. However, our choice (3 years gains) is conservative and the threshold, to be considered as a 3 years life expectancy gain in cardiovascular patients, is high [19, 23]. Moreover, Hage et al. [33] studied physical activity level and health related quality of life three to 6 years after a myocardial infarction in elderly Sweden patients. They concluded that after a coronary event, a supervised physical activity program has the potential to positively influence activity and quality of life for as long as three to 6 years.

## Conclusion

To conclude, our study has shown first, that a physical activity program is cost effective, providing a better quality of life in patients with coronary artery disease or moderate heart failure history, for a reasonable cost corresponding to the health care consumption reduction observed 1 year after the intervention, and second, that a PAPA program is more efficient than a SPA program on quality of life improvement.

## Additional file

Additional file 1: Questionnaires used in the « As du coeur » study. (PDF 558 kb)

## Abbreviations

EQ-5D: European Quality in 5 Dimensions
NYHA: New York Heart Association
PA: Physical activity
PAPA: Progressively Autonomous Physical Activity
QALYs: Quality Adjusted Life Years
SAQ: Seattle Angina Questionnaire
SPA: Supervised Physical Activity
T0: At baseline
T12: At 1 year after the beginning of the intervention (12 months)
T6: After the physical activity program (6 months)
