# Supplementary material for: “As du Coeur” study: a randomized controlled trial on quality of life impact and cost effectiveness of a physical activity program in patients with cardiovascular disease
Source: BMC Cardiovasc Disord. 2018 Dec 6;18:225. doi: 10.1186/s12872-018-0973-3 (PMC6284296; doi:10.1186/s12872-018-0973-3)
Supplement: Supplementary file 1 — Questionnaires used in the « As du coeur » study. (PDF 558 kb) [file 12872_2018_973_MOESM1_ESM.pdf]

Questionnaires used in the « As du cœur » study

Mesure du Qaly (*Quality Adjusted Life Years*)

Le questionnaire EQ-5D-3L

Mobilité

1. Je n'ai aucun problème pour me déplacer à pied.
2. J'ai des problèmes pour me déplacer à pied.
3. Je suis oblige(e) de rester alite(e).

Autonomie de la personne

1. Je n'ai aucun problème pour prendre soin de moi.
2. J'ai des problèmes pour me laver ou m'habiller tout(e) seul(e).
3. Je suis incapable de me laver ou de m'habiller tout(e) seul(e).

Activités courantes

1. Je n'ai aucun problème pour accomplir mes activités courantes
(e.g. travail, études, travaux domestiques, activités familiales ou loisirs).
2. J'ai des problèmes pour accomplir mes activités courantes.
3. Je suis incapable d'accomplir mes activités courantes.

Douleurs/gêne

1. Je n'ai ni douleurs ni gêne.
2. J'ai des douleurs ou une gêne modérée(s).
3. J'ai des douleurs ou une gêne extrême(s).

Anxiété/Dépression

1. Je ne suis ni anxieux (se) ni déprimé(e).
2. Je suis modérément anxieux (se) ou déprimé(e).
3. Je suis extrêmement anxieux (se) ou déprimé(e).

EQ-5D-3L (UK English)

By placing a tick in one box in each group below, please indicate which statements best describe your own health state today.

**Mobility**	
I have no problems in walking about	☐
I have some problems in walking about	☐
I am confined to bed	☐
**Self-Care**	
I have no problems with self-care	☐
I have some problems washing or dressing myself	☐
I am unable to wash or dress myself	☐
**Usual Activities**	
(*e.g. work, study, housework, family or leisure activities)*	
I have no problems with performing my usual activities	☐
I have some problems with performing my usual activities	☐
I am unable to perform my usual activities	☐
**Pain/Discomfort**	
I have no pain or discomfort	☐
I have moderate pain or discomfort	☐
I have extreme pain or discomfort	☐
**Anxiety/Depression**	
I am not anxious or depressed	☐
I am moderately anxious or depressed	☐
I am extremely anxious or depressed	☐

Source :
Mandy van Reenen and Mark Oppe, *"EQ5D User guide*", April 2015, p. 5.

Questionnaire généraliste de Qualité de vie SF 36

Wade JE, Sherbourne CD. The MOS 36-item short-form health survey (SF-36). Medical Care1992;30:473–483.

Madame, Monsieur,
Merci de bien bouloir remplir ce questionnaire en lisant bien les questions et les instructions
Cela permettra d'apprécier votre ressenti par rapport à vos problèmes de santé et de pouvoir prendre en compte r les progrès ou les éventuels problèmes.
Avec tous nos remerciements

QUESTIONNAIRE

1.- En général, diriez-vous que votre santé est : (entourez ce que vous ressentez)
Excellente _ _ Très bonne _ _ Bonne _ _ Satisfaisante _ _ Mauvaise

2.- Par comparaison avec il y a un an, que diriez-vous sur votre santé aujourd'hui par rapport à il y a 1 an ?
Bien meilleure _ Un peu meilleure _ A peu près identique _ Un peu moins bonne _ Pire

3.- Vous pourriez vous livrer aux activités suivantes le même jour. Est-ce que votre état de santé vous impose des limites dans ces activités ? Si oui, dans quelle mesure ? (entourez la flèche).

a. **Activités intenses** : courir, soulever des objets lourds, faire du sport.
_____↓____________________________↓______________________________↓______
oui, très limité oui, plutôt limité pas limité du tout

b. **Activités modérées** : déplacer une table, passer l'aspirateur.
_____↓____________________________↓______________________________↓______
oui, très limité oui, plutôt limité pas limité du tout

c. **Soulever et transporter les achats d'alimentation**.
_____↓____________________________↓______________________________↓______
oui, très limité oui, plutôt limité pas limité du tout

d. **Monter plusieurs étages à la suite.**
_____↓____________________________↓______________________________↓______
oui, très limité oui, plutôt limité pas limité du tout

e. **Monter un seul étage**.
_____↓____________________________↓______________________________↓______
oui, très limité oui, plutôt limité pas limité du tout

f. **Vous agenouiller, vous accroupir ou vous pencher très bas**.
_____↓____________________________↓______________________________↓______

oui, très limité | oui, plutôt limité | pas limité du tout

g. **Marcher plus d'un kilomètre et demi.**
_____↓_____________________________↓_______________________________↓______
oui, très limité oui, plutôt limité pas limité du tout

h. **Marcher plus de 500 mètres**
_____↓_____________________________↓_______________________________↓______
oui, très limité oui, plutôt limité pas limité du tout

i. Marcher seulement 100 mètres.
_____↓_____________________________↓_______________________________↓______
oui, très limité oui, plutôt limité pas limité du tout

j. **Prendre un bain, une douche ou vous habiller**.
_____↓_____________________________↓_______________________________↓______
oui, très limité oui, plutôt limité pas limité du tout

4.- Au cours des 4 dernières semaines, avez-vous eu l'une des difficultés suivantes au travail ou lors des activités courantes, du fait de votre santé ? (réponse : entourez oui ou non à chaque ligne)

Limiter le temps passé au travail, ou à d'autres activités ?	oui	non
Faire moins de choses que vous ne l'espériez ?	oui	non
Trouver des limites au type de travail ou d'activités possibles ?	oui	non
Arriver à tout faire, mais au prix d'un effort	oui	non

5.- Au cours des 4 dernières semaines, avez-vous eu des difficultés suivantes au travail ou lors des activités courantes parce que vous étiez déprimé ou anxieux ? (réponse : oui ou non à chaque ligne).

Limiter le temps passé au travail, ou à d'autres activités ?	oui	non
Faire moins de choses que vous n'espériez ?	oui	non
Ces activités n'ont pas été accomplies aussi soigneusement que d'habitude ?	oui	non

6.- Au cours des 4 dernières semaines, dans quelle mesure est-ce que votre état physique ou mental ont perturbé vos relations avec la famille, les amis, les voisins ou d'autres groupes ?
____↓____________________↓__________________↓_____________________↓_____
_
pas du tout très peu assez fortement
énormément

7.- Avez-vous enduré des souffrances physiques au cours des 4 dernières semaines ?
____↓____________________↓__________________↓_____________________↓_____
_
pas du tout très peu assez fortement

énormément

8.- Au cours des 4 dernières semaines la douleur a-t-elle gêné votre travail ou vos activités usuelles ?

____↓____________________↓___________________↓_____________________↓_____
pas du tout   très peu   assez fortement   énormément

9.- Ces 9 questions concernent ce qui s'est passé au cours de ces dernières 4 semaines. Pour chaque question, donnez la réponse qui se rapproche le plus de ce que vous avez ressenti. Comment vous sentiez-vous au cours de ces 4 semaines :

a. vous sentiez-vous très enthousiaste ?

____↓________________↓______________↓____________↓______________↓__
tout le temps   très souvent   parfois   peu souvent   jamais

b. étiez-vous très nerveux ?

____↓________________↓______________↓____________↓______________↓__
tout le temps   très souvent   parfois   peu souvent   jamais

c. étiez-vous si triste que rien ne pouvait vous égayer ?

____↓________________↓______________↓____________↓______________↓__
tout le temps   très souvent   parfois   peu souvent   jamais

d. vous sentiez-vous au calme, en paix ?

____↓________________↓______________↓____________↓______________↓__
tout le temps   très souvent   parfois   peu souvent   jamais

e. aviez-vous beaucoup d'énergie ?

____↓________________↓______________↓____________↓______________↓__
tout le temps   très souvent   parfois   peu souvent   jamais

f. étiez-vous triste et maussade ?

____↓________________↓______________↓____________↓______________↓__
tout le temps   très souvent   parfois   peu souvent   jamais

g. aviez-vous l'impression d'être épuisé(e) ?

____↓________________↓______________↓____________↓______________↓__
tout le temps   très souvent   parfois   peu souvent   jamais

h. étiez-vous quelqu'un d'heureux ?

____↓________________↓______________↓____________↓______________↓__
tout le temps   très souvent   parfois   peu souvent   jamais

i. vous êtes-vous senti fatigué(e) ?

____↓________________↓______________↓____________↓______________↓__

tout le temps très souvent parfois peu souvent jamais

10.- Au cours des 4 dernières semaines, votre état physique ou mental a-t-il gêné vos activités sociales comme des visites aux amis, à la famille, etc ?

____↓________________↓______________↓____________↓______________↓__

tout le temps très souvent parfois peu souvent jamais

11.- Ces affirmations sont-elles vraies ou fausses dans votre cas ?

a. il me semble que je tombe malade plus facilement que d'autres.

_____↓______________↓________________↓________________↓______________↓_
_

tout à fait vrai assez vrai ne sais pas plutôt faux faux

b. ma santé est aussi bonne que celle des gens que je connais.

_____↓______________↓________________↓________________↓______________↓_
_

tout à fait vrai assez vrai ne sais pas plutôt faux faux

c. je m'attends à ce que mon état de santé s'aggrave.

_____↓______________↓________________↓________________↓______________↓_
_

tout à fait vrai assez vrai ne sais pas plutôt faux faux

d. mon état de santé est excellent.

_____↓______________↓________________↓________________↓______________↓_
_

tout à fait vrai assez vrai ne sais pas plutôt faux faux

COMMENTAIRES EVENTUELS OU REMARQUES

Tests de capacité fonctionnelle

Tests de condition physique validés par la Société Française de Médecine de l'Exercice et du Sport (SFMES)

- **Test des 6 minutes marche** : Le test des 6 minutes mesure la distance parcourue par un patient en six minutes.

  Afin que les évaluations soient comparables d'une évaluation à l'autre, d'un patient à l'autre et d'un centre à l'autre, le parcours sera standardisé de la façon suivante : le parcours devra être parfaitement plat, d'une distance de 50 m que le patient effectuera en aller/retour autour d'un plot ou d'une chaise (la mesure du parcours de 50 m devra tenir compte de la portion nécessaire au retournement).

  Ce test initialement utilisé pour évaluer les capacités d'endurance et à l'effort des participants permet parallèlement l'évaluation des capacités locomotrices.
